# Relationship among total tear IgE, specific serum IgE, and total serum IgE levels in patients with pollen-induced allergic conjunctivitis

**DOI:** 10.1007/s00417-021-05348-0

**Published:** 2021-08-20

**Authors:** Yasuo Yamana, Satoshi Yamana, Eiichi Uchio

**Affiliations:** 1 Yamana Eye Clinic, Fukuoka, Japan; 2 Department of Ophthalmology, Graduate School of Medical Sciences, Kyushu University, Fukuoka, Japan; 3 Department of Ophthalmology, Faculty of Medicine, Fukuoka University, Fukuoka, Japan

**Keywords:** Pollen-induced allergic conjunctivitis, Food-induced allergic conjunctivitis, Total tear IgE, Specific serum IgE, Total serum IgE

## Abstract

**Background:**

Recently, the number of patients with pollinosis, particularly Japanese cedar pollinosis, has markedly increased. We previously reported about local allergic conjunctivitis, which is a phenotype of allergic conjunctivitis (AC). AC cases are often sensitized by various antigens. This study aimed to investigate the relationship among total tear IgE (t-tIgE), specific serum IgE (s-sIgE), and total serum IgE (t-sIgE) levels in patients with pollen-induced AC.

**Methods:**

In 2019, 1372 patients were clinically diagnosed with AC at the Yamana Eye Clinic using t-tIgE, t-sIgE, and s-sIgE tests against 39 allergens. Among the pollen-induced AC patients who underwent allergen testing, 99 tested positives for s-sIgE against pollen. The subjects comprised 33 (33.3%) male and 66 (66.7%) female individuals aged 9–86 years.

**Results:**

The t-tIgE test was positive in 68 (68.7%) patients and negative in 31 (31.3%) patients. In the t-sIgE test, 45 (45.5%) patients had t-sIgE levels above the reference value of 170 IU/mL. The higher the total score of the positive class value of each pollen-specific IgE (pollen-sIgE) antibody, the higher the positive rate of t-tIgE (*p* < 0.001). Of 32 patients in whom food-specific IgE (food-sIgE) was detected, 81.3% of the pollen-sIgE-positive and food-sIgE-positive cases were also positive for t-sIgE and t-tIgE. However, significant difference was not found between the total score of food-sIgE of the t-tIgE positive group and negative group.

**Conclusions:**

Pollen-induced AC is caused by pollen sensitization of the conjunctiva. Food-induced AC might be induced by the different pathological mechanism involved in pollen-induced AC.

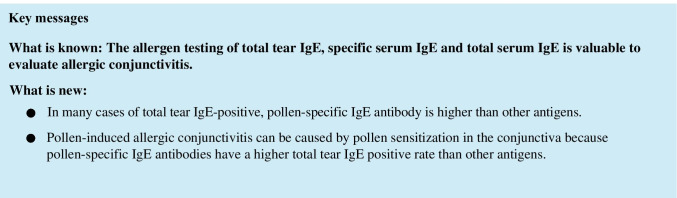



## Introduction

Recently, the number of patients with pollinosis, particularly Japanese cedar pollinosis, has markedly increased [1, 2]. The antigens that cause allergic conjunctivitis (AC), their sensitization pathways, and pathogenic mechanisms are diverse and are not only sensitized to a single antigen but often to multiple antigens. In addition, allergic diseases have various phenotypes such as local allergic rhinitis and asthma [3–5]. Patients with AC are also sensitized to the conjunctiva by various antigens [3]. We previously reported that AC has different phenotypes [6].

Aghayan-Ugurluoglu et al. analyzed timothy grass and birch and found allergen-specific IgA antibodies in the mucosal secretions of patients (e.g., tears) [7]. Ibrahim et al. reported that there was a correlation between specific IgE in tears and skin in patients with AC [8]. In Japan, Mimura et al. reported that both cedar pollen- and *Dermatophagoides pteronyssinus-*specific IgE levels in tears were significantly higher in the allergic group than in the control group [9].

We diagnosed and treated AC according to the Guidelines for the Clinical Management of Allergic Conjunctival Disease of the Japanese Ophthalmology Society (hereafter referred to as “the guidelines”) [10]. This study aimed to investigate the relationship among total tear IgE (t-tIgE), specific serum IgE (s-sIgE) against 39 allergens, and total serum IgE (s-tIgE) levels in patients with pollen-induced AC.

Regarding food-induced AC, the Japanese guidelines for food allergy 2020 [11] only describe symptoms such as conjunctival hyperemia, edema, and pruritus for food-induced AC. Thus, we also investigated the relationship between food- and pollen-induced AC.

## Methods

### Study design and participants

This study followed the ethical principles of the Declaration of Helsinki and was approved by the Ethics Committee of Japan Clinical Society of Diabetes (No. 22/10//2019–6). The study has been registered as the retrospective observational study in the UMIN Clinical Trials Registry (UMIN Trial ID: UMIN000041978). Informed consent was obtained from all individual participants included in this study.

In 2019, 1372 patients obtained a clinical diagnosis of AC based on subjective and clinical symptoms at the Yamana Eye Clinic. The patients who complained of allergic symptoms such as eye itching, discomfort, redness, swelling, eye mucus, nasal discharge, sneezing and stuffy nose, and underwent allergen testing were included. The patients without the results of t-tIgE, s-sIgE, and t-sIgE tests were excluded. There are no age or gender restrictions on the participants.

We have been performing allergen testing for the patients who wish to have them since 2013, and 212 patients underwent allergen testing. Among the 212 patients, 99 (46.7%) tested positive for s-sIgE against pollen. This study included 33 (33.3%) male and 66 (66.7%) female participants aged 9–86 years (Table 1).

**Table 1:** Characteristic of participants (*n* = 99)

Age group (years)	Number (%)	Total serum IgE values (IU/mL)	Number	Specific serum IgE*	Number	Total tear IgE	Number	Sex	Number
< 10	1 (1.0%)	< 170	1	Pollen only	0	Positive	1	Men	1
		≥ 170	0	Other inhaled	1	Negative	0	Women	0
				Food	0				
10–19	5 (5.1%)	< 170	3	Pollen only	1	Positive	3	Men	3
		≥ 170	2	Other inhaled	4	Negative	2	Women	2
				Food	0				
20–29	2 (2.0%)	< 170	0	Pollen only	0	Positive	2	Men	0
		≥ 170	2	Other inhaled	2	Negative	0	Women	2
				Food	2				
30–39	4 (4.0%)	< 170	1	Pollen only	0	Positive	3	Men	1
		≥ 170	3	Other inhaled	4	Negative	1	Women	3
				Food	2				
40–49	6 (6.1%)	< 170	1	Pollen only	1	Positive	5	Men	1
		≥ 170	5	Other inhaled	5	Negative	1	Women	5
				Food	2				
50–59	10 (10.1%)	< 170	7	Pollen only	3	Positive	8	Men	3
		≥ 170	3	Other inhaled	7	Negative	2	Women	7
				Food	3				
60–69	27 (27.3%)	< 170	18	Pollen only	13	Positive	14	Men	10
		≥ 170	9	Other inhaled	14	Negative	13	Women	17
				Food	5				
70–79	31 (31.3%)	< 170	14	Pollen only	8	Positive	22	Men	12
		≥ 170	17	Other inhaled	20	Negative	9	Women	19
				Food	16				
≥ 80	13 (13.1%)	< 170	9	Pollen only	7	Positive	10	Men	2
		≥ 170	4	Other inhaled	6	Negative	3	Women	11
				Food	2				
Total	99	< 170	54	Pollen only	33	Positive	68	Men	33
		≥ 170	45	Other inhaled	63	Negative	31	Women	66
				Food	32				

^*^There are cases where other inhaled specific IgE and food-specific IgE overlap. The list of allergens which were detected was shown in Table 2

### Measurement of allergen testing

The following tests were performed according to “the guidelines.” The t-tIgE levels were measured using the Allerwatch® Tear IgE kit (Wakamoto Pharmaceutical Co., Ltd., Tokyo, Japan) with immunochromatography [12, 13]. The s-sIgE antibody levels against 39 allergens, including *D. pteronyssinus*, cockroach, cat dander, dog dander, Japanese cedar, orchard grass, ragweed, and mugwort, were measured using the View Allergy 39® kit (Thermo Fisher Diagnostics K.K., Tokyo, Japan) with a fluorescence enzyme immunoassay (FEIA) (Table 2). View Allergy 39® kit also utilizes immunochromatography based on a sandwich method but with *β*-galactosidase as the labeled substance. Judgment criteria of View Allergy 39® kit are the following: class 1 is false positive and ≥ 0.27 index; classes 2 to 6 are positive and class 2 is ≥ 0.50 index; class 3 is ≥ 1.80 index; class 4 is ≥ 7.05 index; class 5 is 17.35 index; and class 6 is 29.31 index. The t-sIgE level was measured using FEIA: IgE-RIST (radio-immunosorbent test). The standard value of t-sIgE was < 170 IU/mL and is the reference range that includes 95% of healthy people.

**Table 2:** Allergens included in the specific IgE View allergy 39 test kit

Inhaled and other allergens		Food allergens	
House dust	*Dermatophagoides pteronyssinus, house dust*	Egg	Egg, ovomucoid
Pets	Cat dander, dog dander	Milk	Milk
Insects	Moth, cockroach	Wheat	Wheat
Trees	Japanese cedar, Japanese cypress, Alder, black birch	Means/grains/seeds	Peanuts, soybeans, buckwheat, sesame, rice
Herbs/greases	Orchard grass, ragweed, mugwort, timothy	Crustaceans	Shrimp/lobster, crab
Airborne fungi	*Alternaria*, *Aspergillus*	Fruit	Kiwis, apples, bananas
Fungi and others	*Candida*, *Malassezia*, latex	Fish/meat	Tuna, salmon, mackerel, beef, chicken, pork

### Statistical analysis

Mann–Whitney test and Wilcoxon rank-sum test were used for the comparison of the average between negativity and positivity for t-tIgE. *P* value less than 0.05 was considered to indicate statistical significance. All statistical analyses were carried out using R version 3.5.2 software (R Foundation for Statistical Computing, Vienna, Austria).

## Results

### Participants

The participants were 99 AC patients. The data of 99 participants was shown in Table 1. The mean age was 62.6 years; the positivity of t-tIgE was 68 cases, negative in 31 cases, and t-sIgE values of ≥ 170 IU/mL were 45 cases, t-sIgE values of < 170 IU/mL in 54 cases. Inhaled specific IgE other than pollen was detected in 66 cases, and food-specific IgE (food-sIgE) was detected in 32 cases.

### Result of allergen testing

The pollen-specific IgE (pollen-sIgE) test elucidated 99 positive cases; however, the proportion of cases in which only pollen-sIgE was detected was 33 cases (33.3%). In the remaining 66 cases (66.7%), s-sIgE other than pollen-sIgE was detected.

**Fig. 1:**
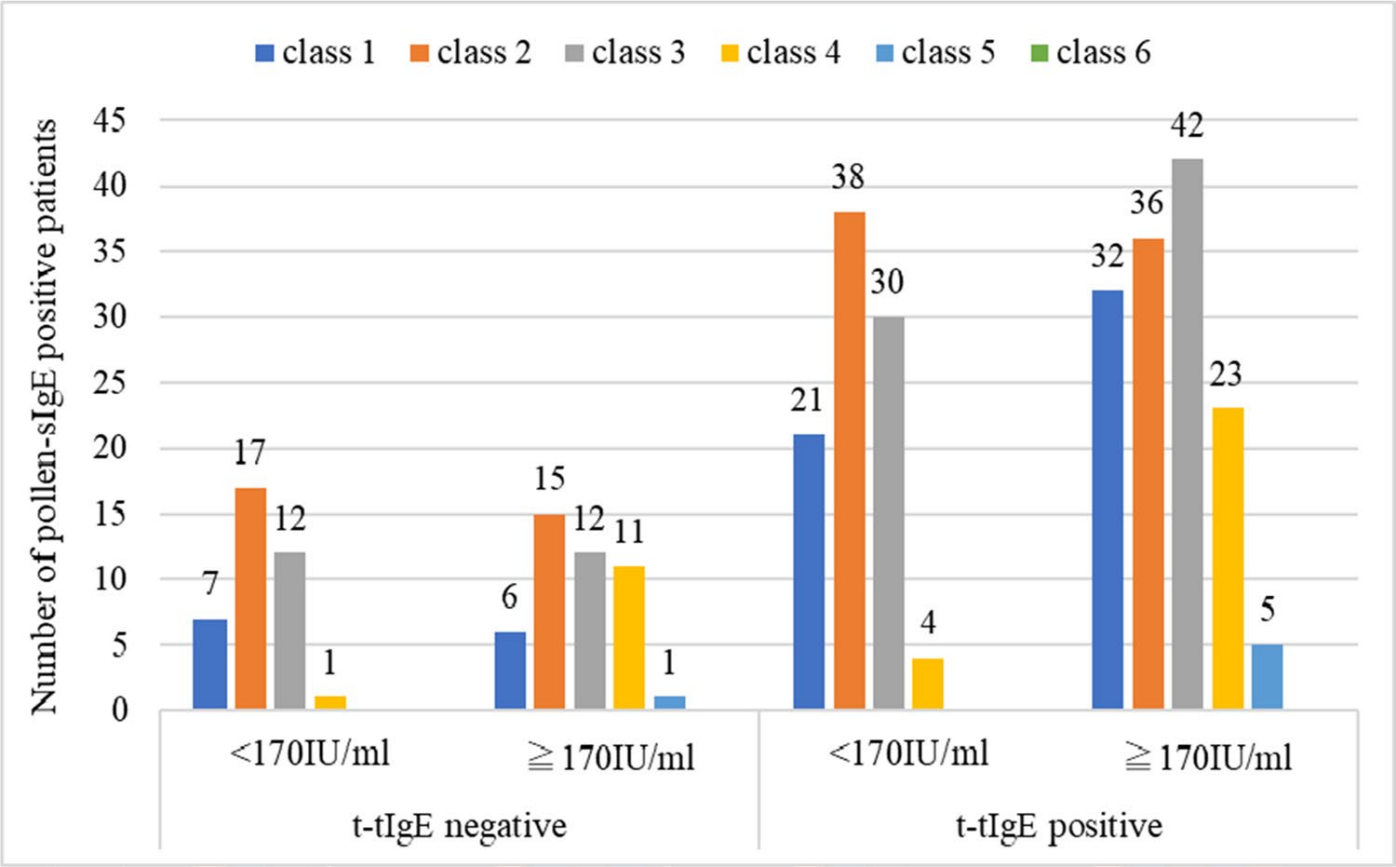
Total serum IgE and total tear IgE (t-tIgE) levels according to the class of pollen-specific IgE (pollen-sIgE). The total serum IgE reference value was less than 170 IU/mL. Class 1 is false positive and ≥ 0.27 index; class 2 is ≥ 0.50 index; class 3 is ≥ 1.80 index; class 4 is ≥ 7.05 index; class 5 is 17.35 index; class 6 is 29.31 index. Classes 2 to 6 are positive

**Fig. 2:**
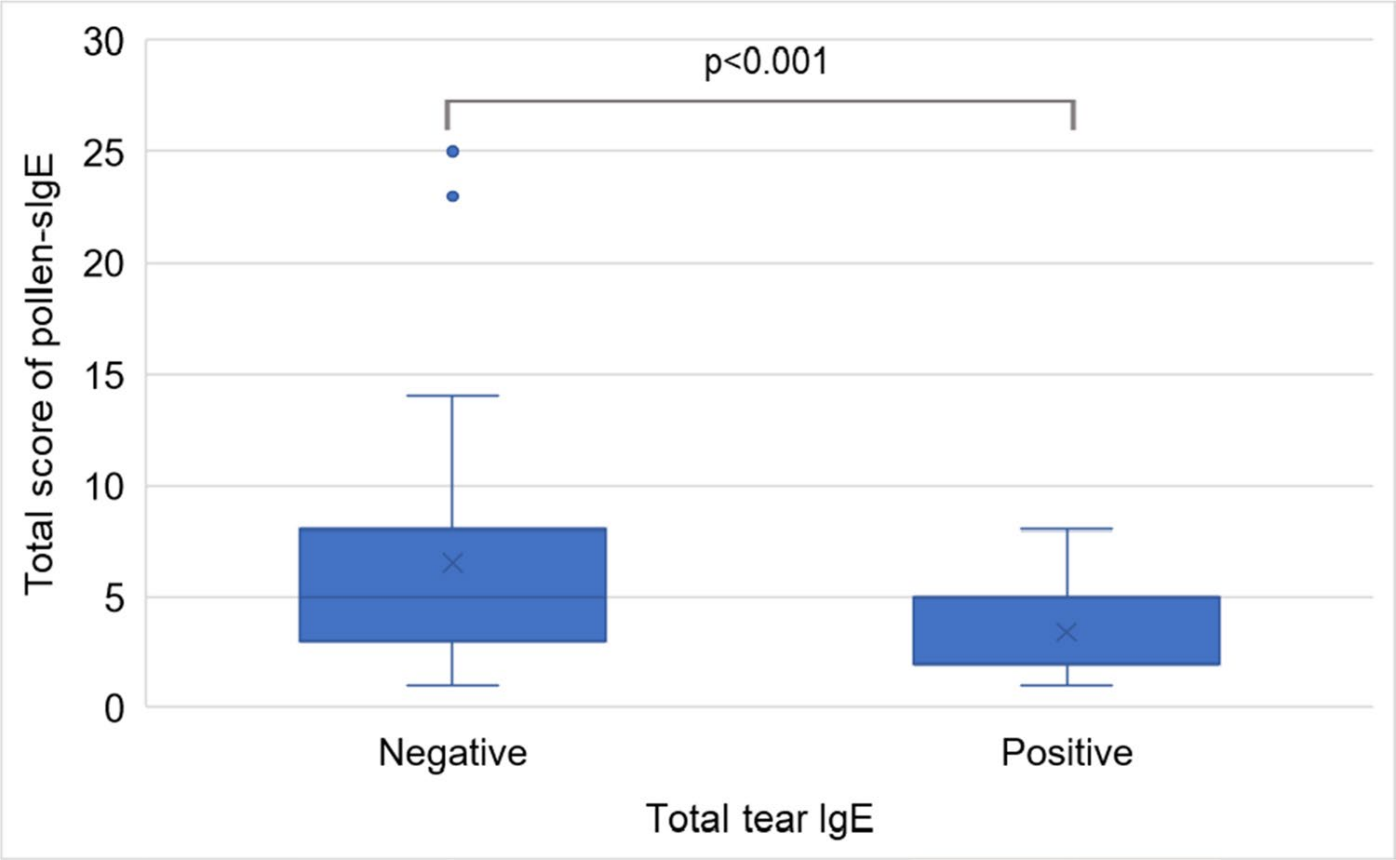
Relationship between the total score of pollen-specific IgE (pollen-sIgE) and total tear IgE levels

Regarding t-sIgE and t-tIgE levels according to the class of pollen-sIgE and regarding t-sIgE and t-tIgE levels of pollen-sIgE for each class, there were many cases in which pollen-sIgE was high among cases in which t-tIgE was positive and t-sIgE was high (Fig. 1). In cases with high total pollen-sIgE score, the positive rate of t-tIgE was also high (*p* < 0.001) (Fig. 2). Their mean ± standard deviation (SD) of the total pollen-sIgE score was 3.4 ± 2.3 points.

The s-sIgE against allergens other than pollen was detected in 66 cases (66.7%). The t-tIgE and t-sIgE test results showed that 68.7% of pollen-sIgE-positive cases were t-tIgE positive, and 45.5% of them had t-sIgE values of ≥ 170 IU/mL. In cases where the t-sIgE value was higher than the standard value, the proportion of cases in which s-sIgE was detected for allergens other than pollen was 93.3%.

**Fig. 3:**
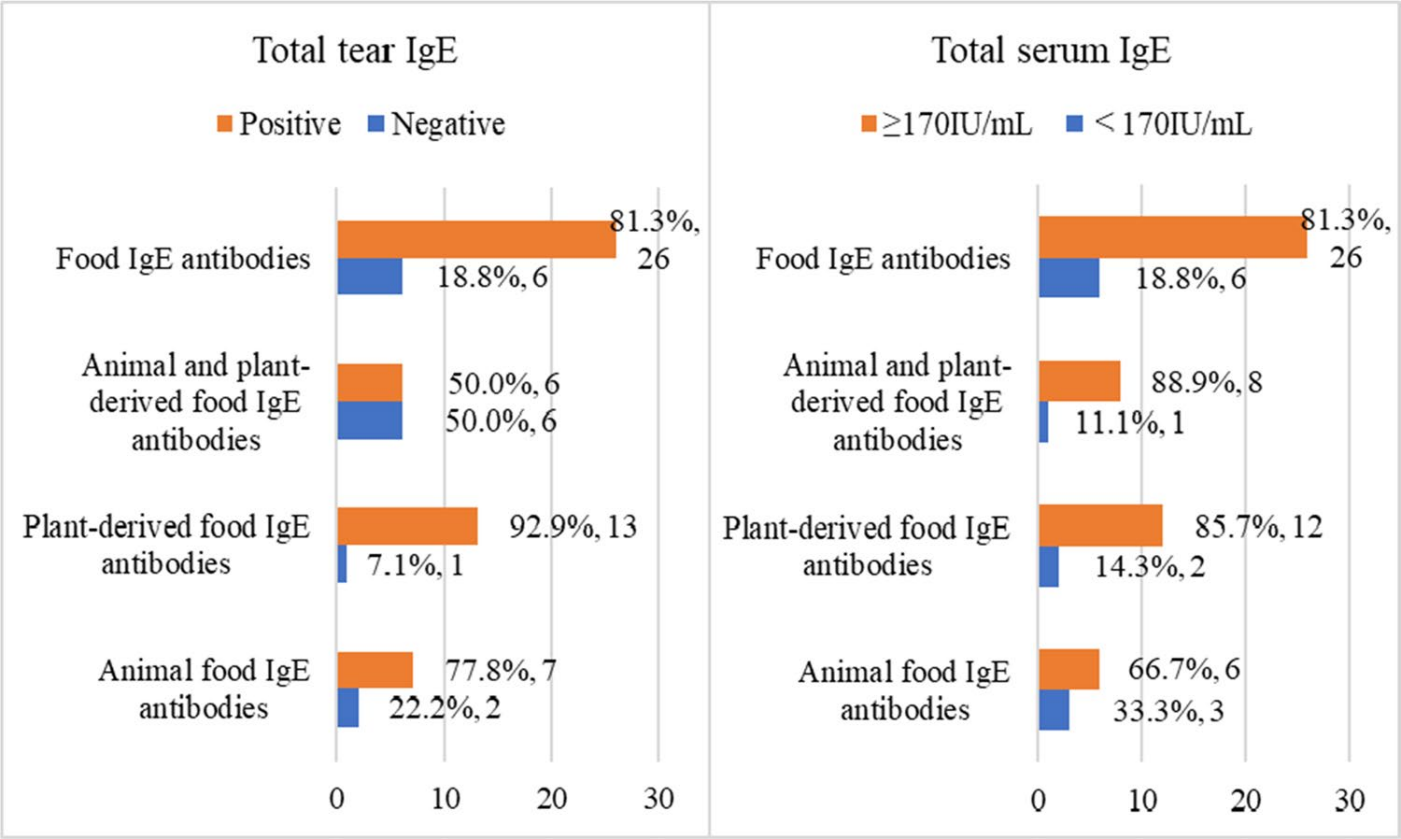
Results of total tear IgE and total serum IgE tests for 32 cases in which food-specific IgE was detected. The total serum IgE reference value was less than 170 IU/mL. Among the cases showing positivity for pollen-specific IgE and food-specific IgE, the total serum IgE and total tear IgE levels were higher than the standard levels in 81.3% of the cases. Animal-derived food IgE includes egg, ovomucoid, milk, shrimp/lobster, crab, tuna, salmon, mackerel, beef, chicken, and pork. Plant-derived food IgE includes wheat, peanuts, soybeans, buckwheat, sesame, rice, kiwis, apple, and bananas

Tables 3 and 4 show the results of t-sIgE and t-tIgE tests for 32 cases in which food-sIgE was detected. In 32 patients who were food-sIgE positive, 81.3% of the pollen-sIgE-positive and food-sIgE-positive cases had t-sIgE and t-tIgE above standard levels (Fig. 3).

**Table 3:** Results of the total serum IgE test for 32 cases in which food-specific IgE was detected

	Total serum IgE	
	< 170 IU/mL	≥ 170 IU/mL
Animal food-specific IgE only	3	6
Plant-derived food-specific IgE only	2	12
Both animal and plant-derived food-specific IgE	1	8
Food-specific IgE	6	26

**Table 4:** Results of the total tear IgE test for 32 cases in which food-specific IgE was detected

	t-tIgE negative	t-tIgE positive
Animal food-specific IgE only	2	7
Plant-derived food-specific IgE only	1	13
Both animal and plant-derived food-specific IgE	3	6
Food-specific IgE	6	26

*t-tIgE*, total tear IgE

**Fig. 4:**
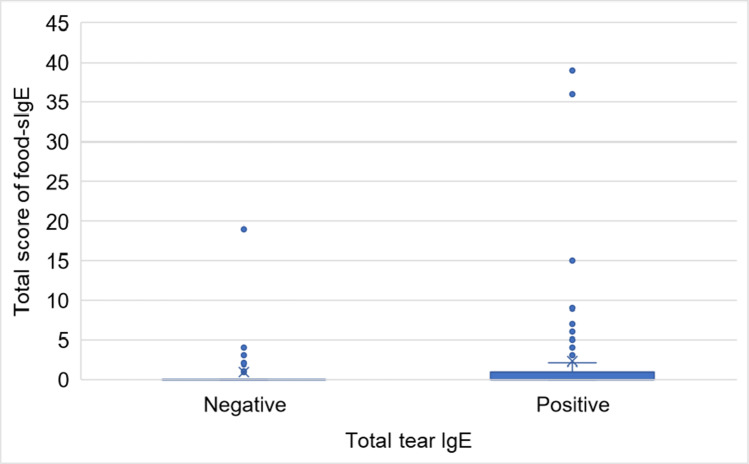
Relationship between the total score of food-specific IgE (food-sIgE) and total tear IgE levels

Regarding the relationship between the total score of food-sIgE and t-tIgE levels, a significant difference was not found between the total score of food-sIgE of the t-tIgE positive group and negative group (Fig. 4).

## Discussion

In “the guidelines” [10], AC is defined as non-proliferative conjunctivitis associated with a type I allergy that is mediated by IgE antibodies. In this study, we examined the relationship among t-tIgE, t-sIgE, and s-sIgE in patients with pollen-induced AC.

A commercial kit, Allerwatch® Tear IgE [12–15], that measures total IgE antibody levels in lachrymal fluids has recently become available, and it confirms the local production of IgE antibodies in the eye.

Aghayan-Ugurluoglu et al. [7] searched for birch and timothy grass pollen allergen-specific IgA antibodies in tear fluids and reported that “serum and tears of many of the pollen-allergic individuals with conjunctivitis exhibited specificity for the very same pollen allergens.”

Ibrahim et al. [8] noted that serum and tears of many pollen-allergic individuals with conjunctivitis exhibited specificity for the same pollen allergens. Hoffmann-Sommergruber et al. [16] investigated whether the presence of IgE in tears of grass pollen-allergic patients correlated with the disease and clinical symptoms. They concluded that allergen-specific IgE antibodies in tears seem to be produced locally rather than as serum exudate and that IgE in tears seems to be responsible for AC. In Japan, Mimura reported that both cedar pollen- and *D. pteronyssinus*-specific IgE levels in tears were significantly higher in the allergic group than in the control group [9]. From these reports, it may be speculated that pollen-induced AC is sensitized by the conjunctiva. In general, allergic patients may be sensitized to multiple antigens to varying degrees. However, until now, no reports have investigated the relationship between various antigens and pollen-induced AC.

Based on the current study results, the t-sIgE positivity rate was as low as 33.3% in the case of pollen-sIgE alone. Moreover, the positivity for both pollen-sIgE and non-pollen-sIgE was 66.7%, which was relatively low. For the t-tIgE positivity rate, even in cases with positivity to only pollen-sIgE, the t-tIgE positivity rate was 63.6%, but only 9.1% of cases had high t-sIgE levels, which was low. Furthermore, for t-sIgE and t-tIgE levels of pollen-sIgE for each class, there were many significant differences among cases in which t-tIgE was positive and t-sIgE was high, and the class of pollen-sIgE was also high.

These results suggest that pollen-induced AC is first sensitized by the conjunctiva. However, the power of sensitization was considered weak with only a single pollen antigen. In addition, it could be inferred that non-pollen-sIgE was sensitized in cases where the t-sIgE level was higher than the standard value.

Next, the relationship between pollen-induced AC and food-induced AC was investigated. Regarding the relationship between food and AC, Mimura et al. [17] stated that wheat allergy is involved in AC. Pollen and food allergies together are known as pollen-food allergy syndrome [18, 19]. This description is not found in the Japanese Food Allergy Guideline 2020 [11], which only explains clinical symptoms such as conjunctival hyperemia and edema.

In this study, 81.3% of pollen-sIgE-positive and food-sIgE-positive cases were associated with t-sIgE and t-tIgE above standard levels. Furthermore, 81.3% of pollen-sIgE-positive and food-sIgE-positive cases showed higher values than the standard t-sIgE and t-tIgE values (Fig. 3). Mimura et al. reported regarding the correlation between the t-tIgE score and s-sIgE levels against house dust mites and found that house dust mite allergens may be the primary cause of AC during autumn in Japan [20]. Despite these reports, the lack of significant difference of the total score of food-sIgE between the t-tIgE positive group and negative group suggests that food-induced AC might be induced by the different pathological mechanism involved in pollen-induced AC.

In conclusion, based on the high proportion of cases with high pollen-sIgE-positive scores among t-tIgE-positive cases, pollen-induced AC is very likely caused by pollen sensitization of the conjunctiva.

## Abbreviations

AC: Allergic conjunctivitis
IgE: Immunoglobulin E
s-sIgE: Specific serum IgE
s-tIgE: Total serum IgE
t-tIgE: Total tear IgE
View39: View allergy 39 kit
RIST: Radio-immunosorbent test
FELA: Fluorescence enzyme immunoassay
pollen-sIgE: Pollen-specific IgE
food-sIgE: Food-specific IgE
D. pteronyssinus: Dermatophagoides pteronyssinus
